# Optimization of flooding parameters and enhanced-efficiency development for surface-modified nano-SiO₂ emulsion in low-permeability sandstone reservoirs

**DOI:** 10.1371/journal.pone.0326805

**Published:** 2025-08-29

**Authors:** Mengke Xin, Dingxue Zhang, Yan Zhang, Jun Chen, Linshuo Yan, Yiwei Qin, Qirui Zhang

**Affiliations:** 1 School of Petroleum Engineering, Yangtze University, Wuhan, Hubei, China; 2 Key Laboratory of Drilling and Production Engineering for Oil and Gas, Hubei Province, Wuhan, Hubei, China; Dawood University of Engineering and Technology, PAKISTAN

## Abstract

Low-permeability reservoirs, characterized by poor reservoir quality and pronounced heterogeneity, consistently encounter engineering challenges during waterflooding operations, such as suboptimal displacement efficiency and restricted sweep coverage. Nanoemulsions, through wettability alteration and enhancing macroscopic sweep efficiency, are widely applied in the efficient development of low-permeability reservoirs. However, current field applications in Reservoir G exhibit inefficacy, necessitating laboratory experiments to identify factors affecting poor injection performance and enhance oil recovery. This study conducted laboratory core displacement experiments using native cores from the target reservoir, integrated field operational data for injection parameter optimization, and implemented field applications. Results indicate: (1) The modified nano-SiO₂ emulsion, with an average particle size of 26.01 nm, is substantially below the pore-throat dimensions of reservoir cores; (2) Wettability reversal was confirmed through contact angle reduction on oil-wet substrates from 132.9° to 53.6°; (3) Optimal parameters—0.1 mL/min injection rate, 0.3 wt% concentration, and 0.3 PV slug volume—Utilizing dual-slug injection (0.15 PV × 2 + 0.1 PV water spacer) enhanced oil recovery by 18.83%. Segmented injection effectively mitigates particle adsorption-induced plugging and achieves deep reservoir penetration, outperforming single-slug injections. Field experimental results show that segmented injection with water spacers increased daily fluid production from 2.86 m³ to 5.22 m³ and daily oil production from 2.23 t to 4.3 t, retaliating a synergistic mechanism of the modified nano-SiO₂ emulsion in “performance-parameter-mode” optimization.

## 1 Introduction

With the rapid development of China’s industrialization and economic growth, energy demand has risen steadily. Current domestic oil and gas production fails to satisfy industrial and infrastructural requirements. Notably, low-permeability reservoirs contain over 60% of China’s hydrocarbon resources, making them crucial targets for field exploration and development [1]. However, low-permeability reservoirs have small pore throats, poor connectivity, very small oil-water flow channels, very high resistance to seepage of fluids, very small elastic energy, and strong forces between liquid-solid interfaces, as well as between liquid-liquid, so that seepage laws generally do not follow Darcy’s law [2–4]. These problems lead to certain difficulties in the development of low-permeability reservoirs, and the realization of the efficient development of this type of reservoir is one of the important problems that need to be solved in the development of oil and gas fields in China. At present, the methods to improve recovery in low permeability reservoirs include polymer flooding, surfactant flooding, foam flooding, gas flooding and so on [5–6]. However, low permeability reservoirs have the problem of water injection challenges or even water injection, and the viscous characteristics of polymers greatly limit their injection capacity in such reservoirs, which further aggravates the risk of reservoir clogging [7–8]; the phenomenon of adsorption loss exists in the process of surfactant flooding, which leads to adsorption and retention on the surface of the rock and reduces the effective concentration of surfactant, which increases the concentration of injection and the cost; the foam flooding is subject to the safety and safety of the foam system in the reservoir, which is a very important issue in the field; Foam flooding due to the presence of foam system in the reservoir security is relatively unstable, making its application in the oilfield is limited; due to the seriousness of the heterogeneity of low-permeability reservoirs, The implementation of gas flooding in low-permeability reservoirs is challenged by gas channeling phenomena and elevated gas sourcing expenditures [8].

Nanomaterials exhibit excellent temperature/salinity resistance, scale effects, and facile surface modification capabilities, making them promising candidates for reservoir development applications [9–12]. Nanoemulsion flooding, an emerging enhanced oil recovery technique, synergizes nanoparticle-assisted chemical flooding with emulsion flooding mechanisms. Silica (SiO₂) nanoparticles—characterized by non-toxicity, odorlessness, and environmental benignancy—are spherical particles (1–100 nm diameter) that have gained prominence in nanoemulsion flooding due to their cost-effectiveness and environmental compatibility [13–14]. It also has excellent anti-aging and corrosion resistance properties, making it particularly suitable for the more demanding environmental conditions in oil and gas reservoirs. Many researchers have conducted studies on enhanced recovery using SiO_2_ nanofluids as oil repellent systems. Zhao et al. [15] synthesized silica Nanoemulsions for low-permeability reservoirs and systematically characterized their physicochemical properties. Experimental results demonstrated that the Nanoemulsions achieved wettability alteration by modifying the oil-wet contact angle from 44.6° to 134.6°. Laboratory displacement experiments revealed that nanoemulsions significantly enhanced the oil-wet contact angle compared to conventional aqueous displacement agents. Furthermore, the nanoemulsions improved crude oil recovery by 18.3% relative to baseline waterflooding performance. Youssif [16] et al. investigated the effect of hydrophilic monodispersed silica nanoparticles with an average particle size of 22 nm on oil recovery by enhanced oil recovery (EOR) processes. The incremental recovery was increased by 13.28% by using 0.1wt% silica nanofluid tertiary recovery technique followed by water-flooding secondary recovery technique. Hendraningrat et al [17] found that nano-silica emulsion can increase the hydrophilicity of the solid surface by reducing the interfacial tension between the oil phase and the water phase. Compared with water flooding, enhanced oil recovery is about 4% ~ 5%. However, nano-SiO₂ exhibits high surface energy and is prone to agglomeration, which restricts its broader application. Surface modification of nano-SiO₂ can significantly mitigate nanoparticle agglomeration and enhance compatibility with polymer molecules, thereby optimizing nanoparticle performance and improving oil displacement efficiency in low-permeability reservoirs [18–21]. Tian et al. [22] modified nano-SiO₂ using a silane coupling agent to study the coupling agent dosage and the performance of the modified nanoparticles. Experimental results demonstrated that the modified nano-SiO₂ system exhibited enhanced wettability alteration and interfacial tension reduction compared to the unmodified system. Cao et al. [19] investigated the effect of surface modification on nano-SiO₂ properties through controlled comparative experiments between modified and unmodified nanoparticles. Experimental results demonstrated that the modified nano-SiO₂ exhibited optimal oil displacement performance, enhancing recovery by 10.3%. Li et al. [23] verified that when the oil-water interfacial tension is not significantly reduced, the surface-modified nanoparticles have a higher ability to separate oil droplets from the surface of oil-wet minerals.

Research both domestically and internationally has shown that pure nano-SiO₂ possesses a high specific surface area, high chemical activity, and exhibits strong surface effects. These properties make nano-particles prone to agglomeration during dispersion, forming clusters that become trapped in the small, narrow pores of rock, thereby reducing the permeability of reservoir rock and limiting their application [24]. Surface modification of nano-silica particles through physicochemical methods enhances their dispersion stability. However, current research predominantly focuses on optimizing the performance of modified nano-SiO₂ emulsions, while inadequately addressing the significant impact of injection parameters—including injection rate, injection volume, injection concentration, and injection mode—on ultimate recovery. This gap results in dual deficiencies in understanding enhanced oil recovery mechanisms and nanoemulsion transport behavior within the deep core matrix. Improper injection methods can easily lead to problems such as particle retention and blockage, premature formation of dominant channels, resulting in poor recovery efficiency and low development efficiency. In addition, injection parameters such as injection rate, injection volume and SiO_2_ nanoparticle concentration of SiO_2_ nanofluids have a great influence on enhanced oil recovery [25]. Block G65 in Oilfield G, with an average porosity of 18.1% and an average permeability of 28.9 mD, is classified as a medium-porosity, low-permeability reservoir. This study focuses on this block, characterizing the fundamental properties of modified nano-SiO₂ emulsion and determining optimal injection parameters through combined injection parameter optimization and dynamic adsorption experiments. The research systematically investigates the effects of injection timing, injection volume, injection rate, injection concentration, and injection method on modified SiO₂ nanoemulsion. Additionally, it elucidates the deep migration mechanisms of modified nano-SiO₂ emulsion within cores, providing theoretical support for field-scale applications.

## 2 Experiments

### 2.1 Materials and equipment

**Fig 1:**
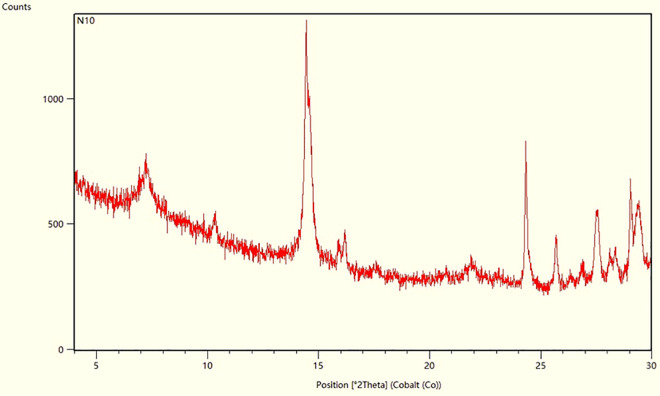
XRD diffraction pattern of the core sample.

Modified nano-SiO₂ emulsion (provided by Jidong Oilfield Research Institute), deionized water, n-heptane (analytically pure, Tianjin Zhiyuan Chemical Reagent Co., Ltd.). The core used in the test is a natural outcrop core from the G reservoir (The XRD diffraction pattern of the core sample is presented in Fig 1, The results of X-ray diffraction whole-rock quantitative analysis are presented in Table 1), with a length of 50 mm and a diameter of 25 mm. The liquid-permeability of the core is 20 × 10 ⁻ ³ μm². Crude oil from the G reservoir has an underground viscosity of 0.92 mPa·s and a density of 0.8241g/cm³. The simulated formation water is laboratory-prepared with a salinity of 4132 mg/L and a NaHCO₃-type water composition. The main ion mass concentrations are as follows: Na⁺ + K ⁺ : 1142 mg/L, Ca² ⁺ : 46 mg/L, Mg² ⁺ : 4 mg/L, Cl ⁻ : 340 mg/L, SO₄² ⁻ : 14 mg/L, HCO₃ ⁻ : 2586 mg/L.

**Table 1:** Results of X-ray diffraction whole-rock quantitative analysis.

Total clay content	Barite	Quartz	K-feldspar	Plagioclase	Calcite	Dolomite	Siderite	Anhydrite
19.6%	0.0%	28.7%	29.0%	15.6%	7.5%	0.6%	0.0%	0.0%

TX-500C Rotating Drop Interfacial Tensiometer (Beijing Pinzhi Chuangsi Precision Instrument Co., Ltd.), DSA30 Optical Contact Angle Measuring Instrument (Kruss, Germany), JK99FM Fully Automatic Interfacial Tensiometer (Shanghai Zhongchen Digital Technology Equipment Co., Ltd.), LC-ES-60 Cantilever Electric Stirrer (Shanghai Li-Chen Bangxi Instrument Co., Ltd.), Multi-functional Large-Size Core Displacement Device (Jiangsu Bocheng Energy Technology Co., Ltd.), SPM-28160 UV-Vis-NIR Spectrophotometer (Hangzhou Yuanfang Optoelectronic Information Co., Ltd.).

### 2.2 Experimental methods

#### 2.2.1 Compatibility Evaluation of Modified Nano-SiO₂ Emulsion.

(1) Compatibility with Formation Water

    Based on the stratigraphic conditions of the target reservoir, modified nano-SiO₂ emulsions with mass fractions of 0.1 wt%, 0.3 wt%, and 0.5 wt% were prepared using simulated formation water. The morphological changes of the mixture of modified nano-SiO₂ emulsions and formation water were observed after high-temperature aging and static treatment.

(2) Rock compatibility

 The effects of formation water and modified nano-SiO₂ emulsions with different concentrations on permeability were compared and analyzed via core displacement experiments. The experimental steps are as follows: First, the core was vacuum-saturated with formation water. Then, formation water flooding and nanoemulsion flooding were conducted sequentially under a constant flow rate of 0.1 mL/min (injection pore volume multiple: 2 PV). The initial permeability of the core was 20.0 ± 0.8 mD at 25 °C. The degree of core damage was calculated using Equation (1).


$$E=\frac{K_{O}-K}{K_{O}}\times 100\%$$ (1)

Where: E—Permeability damage rate of the core, %; K_O_—Initial liquid-phase permeability of the core, 10^−3^μm [2]; K—Permeability of the core at the corresponding replacement volume multiplier, 10^−3^ μm [2].

#### 2.2.2 Evaluation of basic properties of modified nano-SiO₂ emulsion.

(1) Particle Size Distribution: The particle size and particle size distribution of 0.3 wt% nanoemulsion were determined using an Anton Paar Litesizer 500 nanoparticle size analyzer via the dynamic light scattering (DLS) method. The average value was obtained as the final result from multiple measurements.
(2) Interfacial Tension: The oil-water interfacial tension was measured using a Texas-500C spinning drop interfacial tensiometer at 80 °C and 8000 r/min via the spinning-drop method.
(3) Wettability: A quartz sheet was used to simulate an oil-wet rock surface, and the contact angles of modified nano-SiO₂ emulsions with different concentrations were measured via the bubble-trap method using a DSA30 optical contact angle measuring instrument.

#### 2.2.3 Injection parameter optimization experiments.

**Fig 2:**
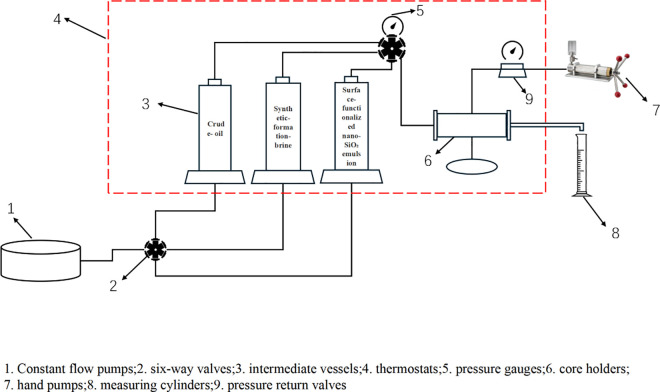
Flow Chart of Core Flooding Experiment.

**Fig 3:**
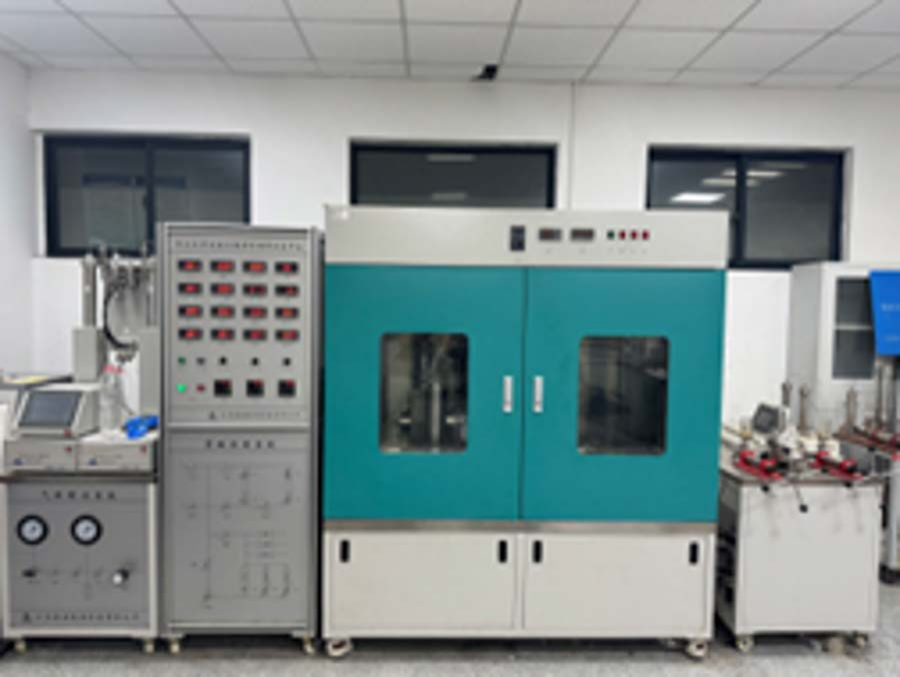
Core Flooding Equipment.

Through indoor displacement experiments, the influence of different injection parameters on the EOR effect in low-permeability reservoirs was characterized, and the optimal injection method and parameters were screened out. The specific experimental steps are as follows: ① Dry the core and weigh its dry weight, vacuum-saturate it with formation water for 24 h, weigh its wet weight, and determine the pore volume; ② Saturate with crude oil, age the core for 48 h, and calculate the original oil saturation; ③ Perform water flooding until no more oil is produced (water cut ≥ 98%) and calculate the original water flooding recovery factor; ④ Conduct optimization experiments on injection parameters and injection methods. Throughout the process, the temperature was maintained at 85 °C, the oil saturation rate was 0.1 mL/min, and the optimal injection parameters were screened using influencing factors including injection rate (0.1, 0.2, 0.3 mL/min), injection concentration (0.1, 0.3, 0.5 wt%), and injection volume (0.1, 0.3, 0.5 PV). The flow chart of the core displacement experiment is shown in Fig 2, and the core displacement equipment is shown in Fig 3.

#### 2.2.4 Dynamic adsorption and retention experiments.

(1) Establishment of Concentration-Absorbance Standard Equation for Surface-Modified Nano-SiO₂ Fluid

Adsorption/retention losses of modified nano-SiO₂ emulsions in porous media are primarily attributed to adsorption on porous media surfaces. This dynamic adsorption experiment quantifies total adsorption/retention by measuring surface adsorption of modified nano-SiO₂ particles [26]. According to the Lambert-Beer law, within specific wavelength ranges, a substance’s absorbance exhibits a linear relationship with its concentration [27]. Leveraging this principle, UV spectrophotometry determines concentration changes through absorbance measurements. UV-Vis spectrophotometry establishes a concentration-absorbance standard curve, yielding a linear regression equation correlating absorbance with concentration.

Dynamic adsorption characteristics of the modified nano-SiO₂ emulsion under different flow rates (0.1 mL/min, 0.2 mL/min, 0.3 mL/min) and different injection modes (single slug injection and 1 PV water-flooded segmented slug injection) were investigated via an indoor core displacement experimental system. Using simulated formation water as the reference blank, the experiment injected a fixed volume of 5 PV of 0.3 wt% surface-modified nano-SiO₂ emulsion followed by 5 PV of simulated formation water. Effluent samples were continuously collected at equal-volume intervals (0.5 PV) from the outlet end to determine average concentration profiles versus injected pore volumes. Concentrations of modified nano-SiO₂ emulsion in the effluent were quantified by UV-Vis spectrophotometry.

## 3 Results and discussion

### 3.1 Compatibility

(1) Compatibility with Formation Water

**Fig 4:**
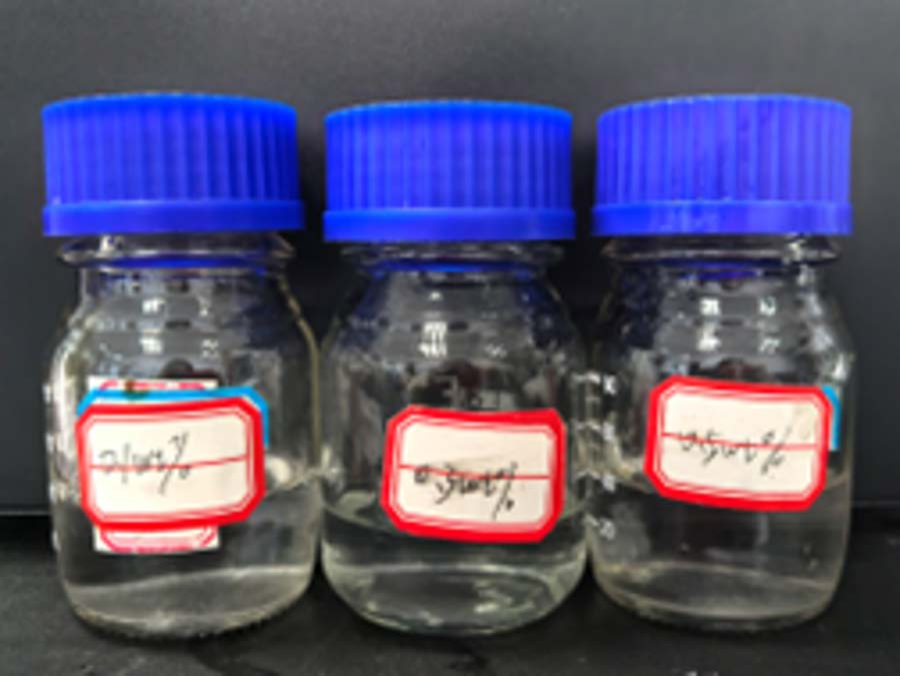
Modified nano-SiO₂ emulsion mixed with formation water before heating.

**Fig 5:**
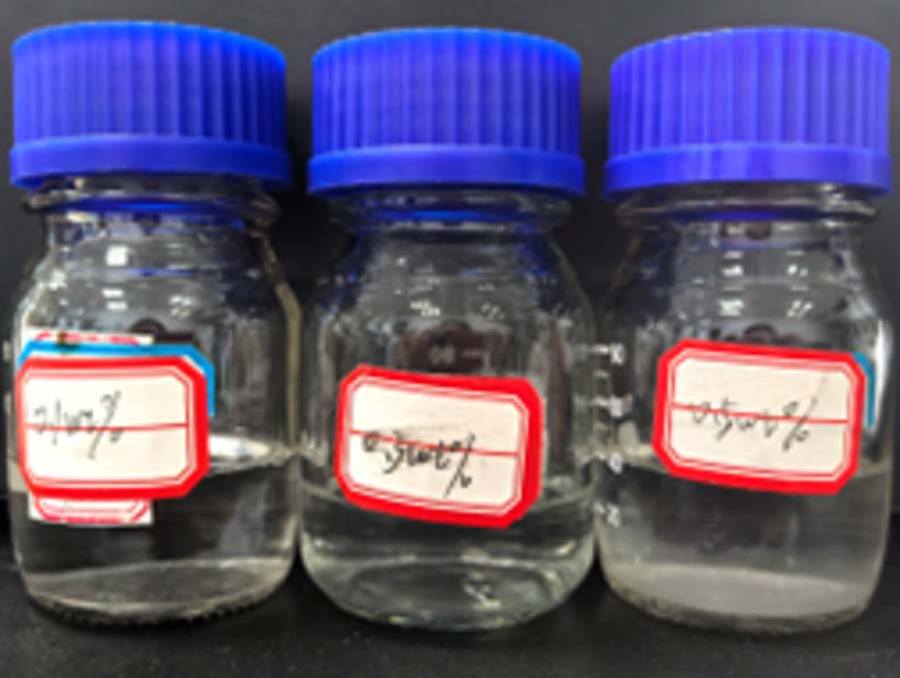
After 48 hours of heating.

After the chemical oil-displacement agent is injected into the formation, it tends to react with formation water to generate precipitates, which block the pore-throat channels of the reservoir and directly affect reservoir integrity and development benefits. To ensure efficient oil and gas field development and achieve reservoir protection and mining process optimization, conducting compatibility evaluation between chemical agents and formation water is of significant engineering importance. In the experiment, modified nano-SiO₂ solutions with mass fractions of 0.1 wt%, 0.3 wt%, and 0.5 wt% were mixed with formation water. After high-temperature aging for 48 hours, observations (as shown in Fig 4 and Fig 5) showed that the different concentration systems remained uniform and stable, without flocculation, precipitation, or phase separation. This confirms that the modified nano-SiO₂ emulsion exhibits excellent compatibility with the target formation water, effectively mitigating the risk of reservoir damage caused by chemical precipitation.

(2) Compatibility with Rocks

**Fig 6:**
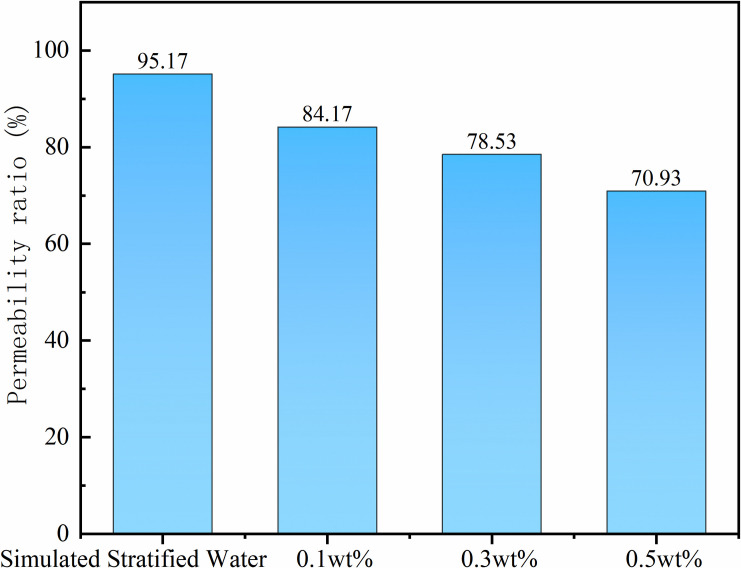
Core damage evaluation diagram.

During chemical flooding, the interaction between reservoir clay minerals and chemical agents readily induces physicochemical reactions such as hydration swelling, particle dispersion, and migration, leading to reservoir permeability damage. Core flow experiment results in Fig 6 show that at a cumulative nanoemulsion injection volume of 2 PV, the core damage rates for the modified nano-SiO₂ emulsion at concentrations of 0.1 wt%, 0.3 wt%, and 0.5 wt% are 15.83%, 21.47%, and 29.07%, respectively. Mechanistic investigations reveal that the emulsion system exhibits excellent compatibility with the rock matrix. When combined with prior formation water compatibility test results (e.g., Fig 6), the engineering application potential of the modified nano-SiO₂ emulsion system for reservoir protection is further validated.

### 3.2 Properties of nanoemulsion

#### 3.2.1 Particle size.

**Fig 7:**
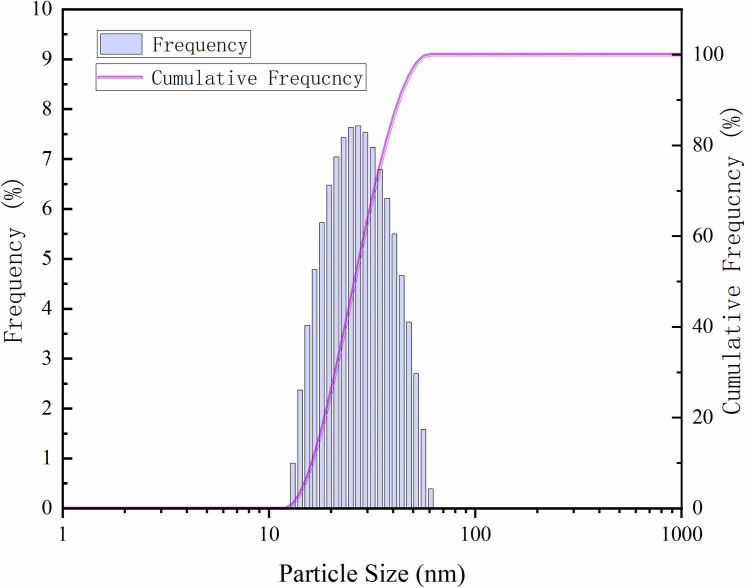
Particle size distribution curve of 0.3 wt% modified nano-SiO₂ emulsion.

**Fig 8:**
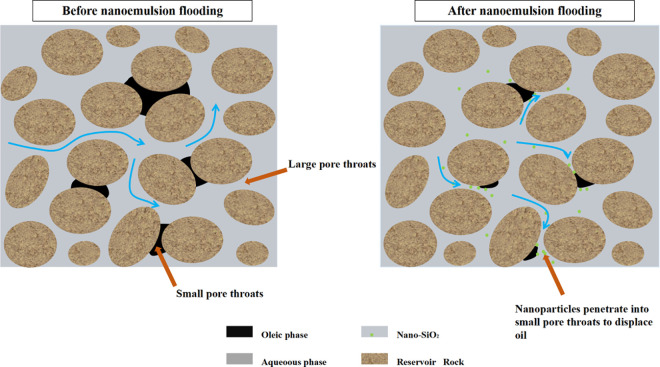
Mechanistic diagram of nanoemulsion displacing oil in small pore throats.

The microstructure characteristics of 0.3 wt% modified nano-SiO₂ emulsion were systematically characterized by laser particle size analysis. As shown in Fig 7, the emulsion system exhibits a single-peak normal distribution in particle size, mainly distributed within the range of 13–62 nm, strictly falling within the nanoscale (median particle size: 26.01 nm). The particle size distribution is narrow and follows a normal distribution pattern. The minimum pore-throat diameter of natural cores is 3–4.5 μm. Analysis indicates that the diameter of modified nanoparticles is only 0.29%–1.38% of the minimum pore-throat size. This scale effect ensures the effective migration of nanoparticles within the pore network, providing a physical basis for the full contact between the emulsion and rock interfaces, as well as the realization of oil-displacement efficiency (Fig 8).

#### 3.2.2 Interfacial tension.

**Fig 9:**
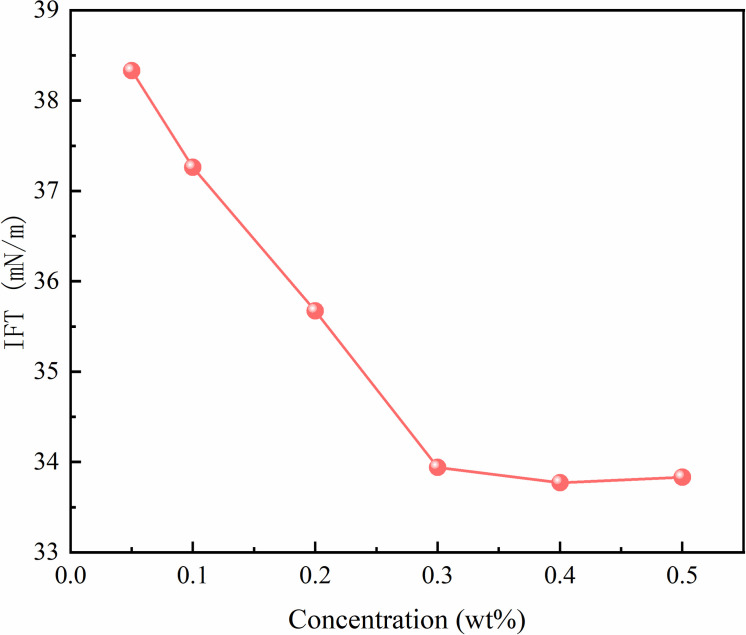
Oil/Water Interfacial Tension of Nano-SiO₂ Emulsions at Different Concentrations.

The interfacial tension between modified nano-SiO₂ emulsion and crude oil at different concentrations is shown in Fig 9 As the nanoemulsion concentration increases from 0.05 wt% to 0.4 wt%, the oil-water interfacial tension decreases with increasing concentration. When the concentration exceeds 0.3 wt%, the interfacial tension no longer decreases but tends to stabilize or exhibit a slight increase. Prior research demonstrates that the primary mechanism for interfacial tension reduction involves the migration and alignment of nanoparticles at the oil-water interface, governed by their hydrophilic-lipophilic properties [28–29]. Furthermore, increasing nanoparticle concentration may cause interfacial tension to initially decrease then increase, potentially exceeding intrinsic interfacial tension values [30–31]. With the increase in modified nano-SiO₂ particle concentration, the adsorption amount at the oil-water interface increases significantly, effectively enhancing interfacial activity and reducing interfacial tension. When the particles form a saturated adsorption layer at the interface, the interfacial tension reaches its minimum value.

#### 3.2.3 Contact angle.

**Fig 10:**
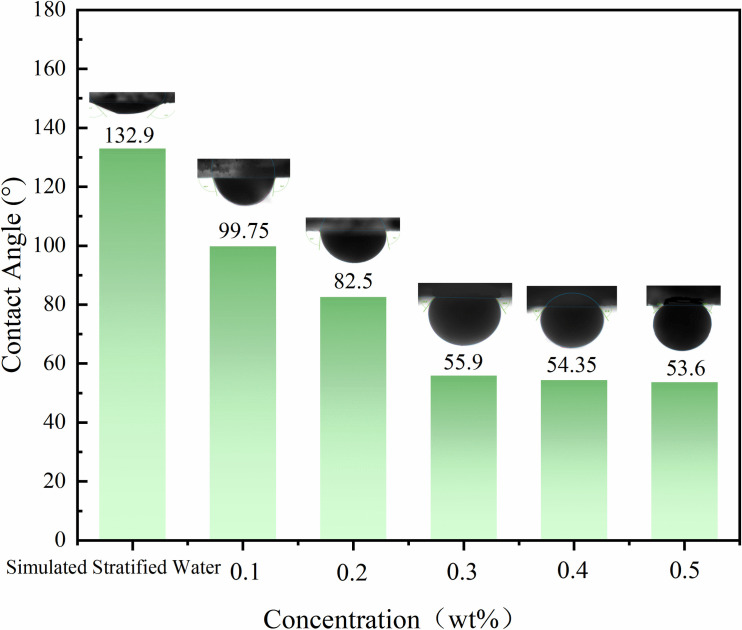
Comparison of Contact Angles for Modified Nano-SiO₂ at Different Concentrations.

Moghaddam et al. [32] identified SiO₂ and CaCO₃ nanoparticles as highly effective wettability agents for regulating the wettability of wellbores and reservoir rocks, with rock wettability serving as a critical geological attribute that directly governs fluid flow behavior and distribution characteristics within porous media. Given that undeveloped reservoirs predominantly exhibit oil-wet or mixed-wet characteristics due to prolonged crude oil impregnation [33], this study employed surface modification technology to render quartz substrates oil-wet. The wettability state was verified by contact angle testing (simulated formation water contact angle: 132.9°), establishing an experimental system equivalent to the actual oil-wet reservoir environment. After treatment with modified nano-SiO₂ emulsions at mass fractions of 0.1wt%, 0.2wt%, 0.3wt%, 0.4wt%, and 0.5wt%, the contact angles of quartz sandstone slices decreased to 99.75°, 82.5°, 55.9°, 54.35°, and 53.6°, respectively (Fig 10), indicating enhanced water-wetness with increasing concentration. This phenomenon aligns with the mechanism observed by Lu et al. [34] via transmission electron microscopy (TEM), wherein nano-SiO₂ adsorption on sandstone surfaces altered pore-wall wettability. It confirms that nanoparticles in modified nano-SiO₂ emulsions, owing to their high specific surface area and surface activity, can tightly adsorb onto active sites on rock surfaces to form an adsorption layer. This facilitates wettability reversal and promotes oil detachment [35–36]. At an emulsion concentration of 0.2wt%, rock wettability reversed from oil-wet to water-wet. Notably, when the concentration exceeded 0.3wt%, the decline rate of contact angles significantly leveled off.

### 3.3 Injection parameter optimization

#### 3.3.1 Injection rate optimization.

**Fig 11:**
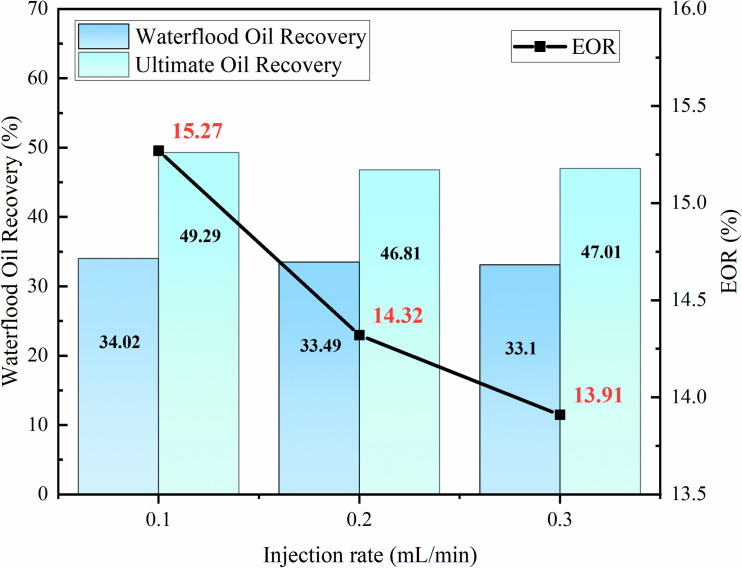
Effect of modified nano-SiO₂ emulsion injection rate on oil recovery.

Through indoor experiments, the EOR increments of modified nano-SiO₂ emulsion under three different injection rates (0.1 mL/min, 0.2 mL/min, 0.3 mL/min) were compared to optimize the best injection rate. Fig 11 shows the oil-displacement effect of modified nano-SiO₂ emulsion with an injection concentration of 0.3 wt% and injection volume of 0.3 PV at different injection rates. As indicated in Fig 11, an inverse correlation exists between the injection rate and oil-displacement efficiency. This is because an increase in injection rate accelerates the movement of the nanoemulsion in the fluid, reducing its contact time with the rock wall. Consequently, the adsorption of nanoemulsion on the rock surface is weakened, causing oil to flow out rapidly and form flow channels in a short time, which easily induces fingering phenomena. As a result, subsequent displacement fluids primarily flow along these seepage channels, while the remaining oil in surrounding pores cannot be effectively displaced, leading to a decrease in recovery. Therefore, the optimal injection rate for the modified nano-SiO₂ emulsion is selected as 0.1 mL/min.

#### 3.3.2 Injection volume optimization.

**Fig 12:**
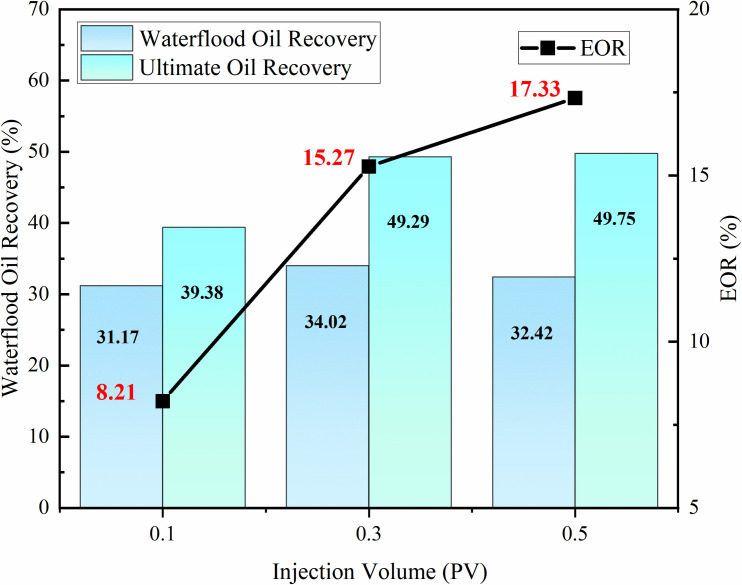
Effect of Modified Nano-SiO₂ Emulsion Injection Volume on Oil Recovery.

Indoor core flooding experiments were conducted using modified nano-SiO₂ emulsion at three different injection volumes (0.1 PV, 0.3 PV, 0.5 PV) to optimize the optimal injection volume. Fig 12 shows the oil displacement efficiency of the modified nano-SiO₂ emulsion with an injection concentration of 0.3 wt% and injection rate of 0.1 mL/min at different injection volumes. When the injection volume increased from 0.1 PV to 0.3 PV, the recovery factor increment was 7.06%; when increased from 0.3 PV to 0.5 PV, the increment was 2.06%. The recovery factor increment increased with the injection volume, but the rate of increase slowed down after exceeding 0.3 PV. Considering the practical reservoir conditions, 0.3 PV was selected as the optimal injection volume, which could enhance the recovery factor by 15.27% compared to water flooding.

#### 3.3.3 Injection concentration optimization.

**Fig 13:**
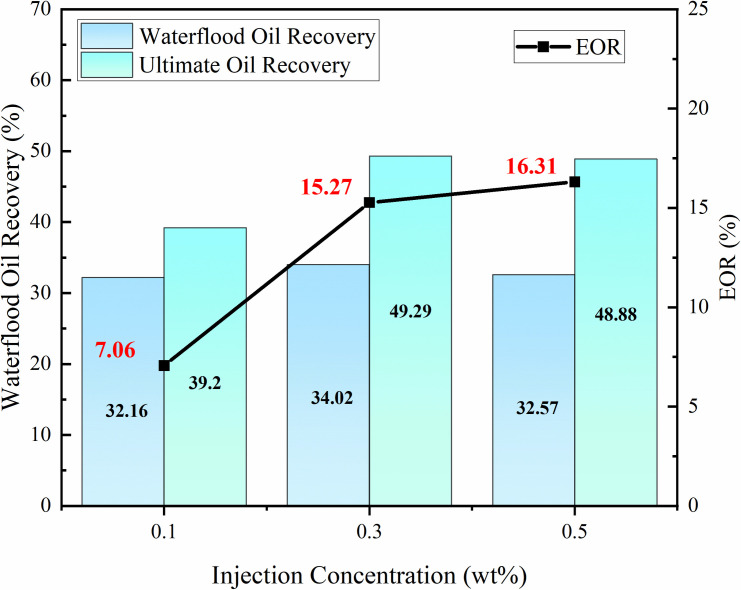
Effect of Modified Nano-SiO₂ Emulsion Injection Concentration on Oil Recovery.

Through three sets of indoor displacement experiments, the EOR increments at different injection concentrations (0.1 wt%, 0.3 wt%, 0.5 wt%) were compared to optimize the best injection concentration. The effect of modified nano-SiO₂ emulsion concentration on EOR is shown in Fig 13 under an injection rate of 0.1 mL/min and injection volume of 0.3 PV. As the nanoemulsion concentration increases, the recovery factor first rises and then increases slowly. This is because when the concentration of the modified nano-SiO₂ emulsion exceeds 0.3 wt%, excessive particle adsorption on the rock surface significantly narrows the seepage channels, inhibiting crude oil migration efficiency and weakening the improvement of recovery factor. Therefore, the optimal injection concentration of the nanoemulsion is selected as 0.3 wt%.

#### 3.3.4 Optimization of injection mode.

**Fig 14:**
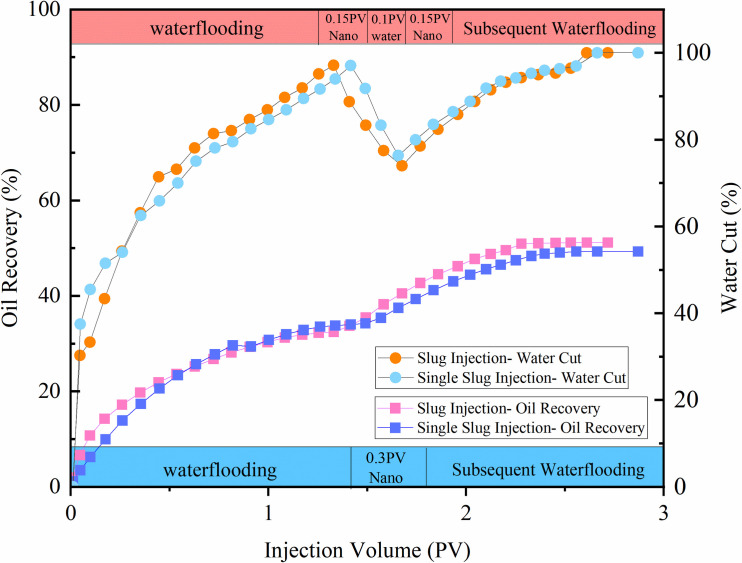
Effect of modified nano-SiO2 emulsion injection mode on oil recovery.

The effect of different injection methods of modified SiO₂ nanoemulsion (single-slug: water flooding + 0.3 PV nanoemulsion flooding + subsequent water flooding; multi-slug: water flooding + 0.15 PV nanoemulsion flooding + 0.1 PV (interval) water flooding + 0.15 PV nanoemulsion flooding + subsequent water flooding) on oil recovery was investigated through two groups of core flooding experiments. The influence of injection methods on enhanced EOR – under conditions of 0.1 mL/min injection rate and 0.3 PV modified nano-SiO₂ injection volume – is shown in Fig 14. The multi-slug injection achieved 18.73% EOR, demonstrating superior performance compared to single-slug injection (15.27%). This improvement stems from the segmented injection method decelerating nanoemulsion transport velocity while prolonging rock surface adsorption time, thereby facilitating deep fluid penetration and enhancing residual oil mobilization in pore-throat blind ends.

### 3.4 Influence factors of dynamic adsorption hysteresis flow rate

Through dynamic adsorption hysteresis experiments, the mechanism of modified nano-SiO₂ action within cores was further verified, supporting its deep transport behavior in core-scale porous media.

#### 3.4.1 Injection rate.

**Fig 15:**
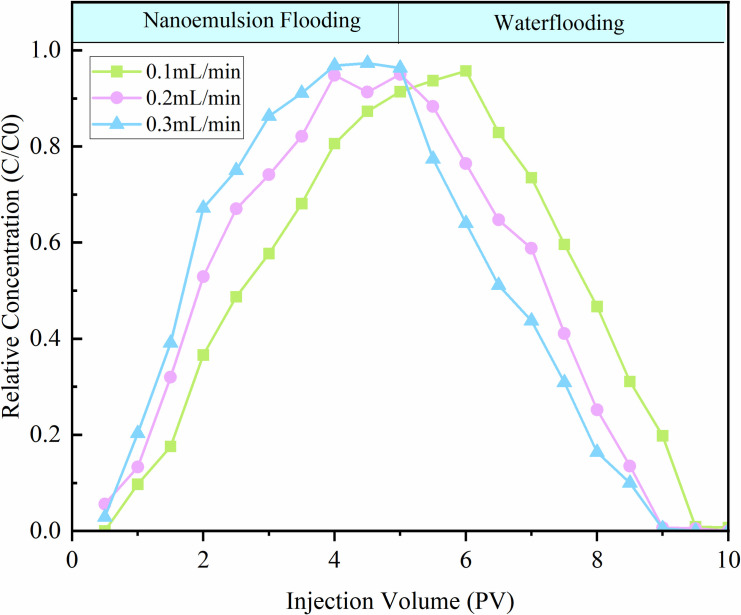
Dynamic adsorption curves at different injection rates.

To investigate the effect of modified SiO₂ nanoemulsion injection rate on its dynamic adsorption in tight reservoirs, the experimental results are presented in Fig 15. A total of 10 PV of effluent was collected during the experiment. As the injected pore volume (PV) of the modified SiO₂ nanoemulsion increased, the relative concentration curve first rose and then plateaued, reaching an inflection point. During the subsequent waterflooding stage, the relative concentration curve declined with an increasing injection rate. Evidently, as the injection rate increased, the effluent concentration reached its peak earlier. This is because a lower injection rate allows for longer contact time between the nanoemulsion and the rock surface, leading to increased nanoemulsion adsorption and enabling the nanoemulsion to more effectively displace oil within the core.

#### 3.4.2 Injection methods.

**Fig 16:**
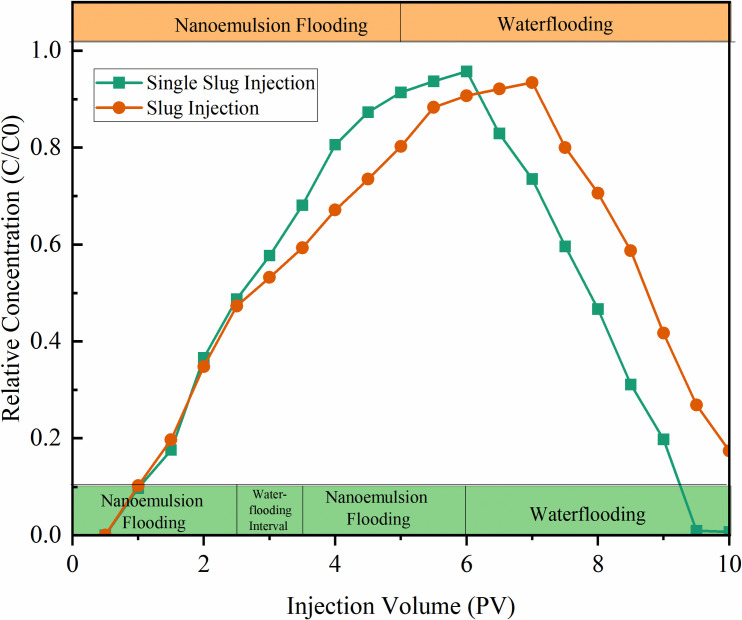
Dynamic adsorption curves under different injection methods.

The effects of different injection modes on the dynamic retention of modified SiO₂ nanoemulsion were investigated, and the experimental results are shown in Fig 16. Experiments revealed that compared with single slug injections, segmented slug injection with 1 PV waterflood interval increased the nanoemulsion retention time in the core. This is because a single large-volume nanoemulsion injection forms a concentrated displacement front in the pore medium, rapidly penetrating high-permeability channels and causing an earlier peak concentration. In contrast, segmented slug injection disperses the nanoemulsion across a broader pore space, slowing the advance of the fluid front and delaying the peak. After each slug injection, the nanoemulsion adsorbs or retains with crude oil and water phases in the pore medium, with part of the emulsion trapped in low-permeability regions. Subsequent slugs must overcome the resistance from prior retention, gradually pushing the emulsion toward the production end and prolonging overall transport time. Thus, multi-stage slug injections effectively extend nanoemulsion transport time in the reservoir by dispersing flow paths, and this delay highlights the potential advantages of multi-stage slug injection in enhancing displacement efficiency and expanding swept volume.

### 3.5 Field application

The primary oil-producing wells in the target block exhibit development characteristics of low liquid production, low water cut, and low reservoir energy. According to optimized parameters from the development plan, enhanced oil recovery field tests using surface-modified nano-SiO₂ emulsion were implemented in Wells G65-42 and G65-43. Both wells demonstrated significant post-treatment responses with clear effectiveness (See Table 2). Specifically, Well G65-42 achieved initial daily incremental liquid production of 2.36 m³ and daily incremental oil production of 2.07 tonnes through segmented waterflooding with intermittent injection, showing remarkable oil increase. Well G65-43 yielded initial daily incremental liquid production of 2.12 m³ and daily incremental oil production of 1.87 tonnes under single-slug injection. Comparative analysis confirms that segmented intermittent waterflooding outperforms single-slug injection with superior incremental production performance.

**Table 2:** Field application performance of surface-modified nano-SiO₂ emulsion flooding.

Well ID	Injection Mode	Pre-Injection		Post-Injection	
		Daily Oil Production/t	Composite Water Cut/%	Daily Oil Production/t	Water Cut/%
G65-42	Single-Slug Injection	2.23	28.1	4.3	20.8
G65-43	Segmented Slug Injection	2.47	30	4.34	25.4
